# Insights into cow-level risk factors for highly pathogenic avian influenza H5N1 clinical disease in lactating Holsteins from a single outbreak in Colorado: An observational study

**DOI:** 10.3168/jdsc.2025-0799

**Published:** 2025-09-10

**Authors:** A. Rico, B. Nicks, A. Lago, N. Silva-del-Rio

**Affiliations:** 1 Department of Population Health and Reproduction, University of California–Davis School of Veterinary Medicine, Davis, CA 95616; 2 University of California–Davis Veterinary Medicine Teaching and Research Center, Tulare, CA 93274; 3 Jager Ag LLC, Eaton, CO 80615; 4 DairyExperts Inc., Tulare, CA 93274

## Abstract

Highly pathogenic avian influenza (HPAI) H5N1 (clade 2.3.4.4b) has spilled over into dairy cattle populations in the United States. A year after the initial outbreak, many uncertainties remain about the virus' epidemiology. The objective of this observational study was to examine cow-level factors that may influence cow's susceptibility to manifest clinical signs during an HPAI H5N1 outbreak using on-farm herd records. The study was conducted on a commercial dairy farm in Colorado that housed only mid-to-late lactation cows and experienced the peak of its outbreak in late May 2024. Herd records were used to identify clinical cases. The study population comprised 3,281 cows that were housed across 7 freestalls and 5 open-lot pens. Pens were classified as comparable if they housed cows with similar individual-level characteristics, as determined using a network-based clustering approach based on ANOVA and chi-squared tests. Univariate associations between case status and potential risk factors were evaluated in the full study population using *t*-tests and chi-squared tests. The association between clinical disease and pregnancy was assessed using a 1:1 matched subset of pregnant and nonpregnant cows (n = 196), matched on parity, lactation stage, milk yield, and pen ID, and analyzed using conditional logistic regression. Other potential risk factors of clinical disease were evaluated in a subset of pregnant cows from comparable pens (n = 1,546) using mixed-effects logistic regression. The overall proportion of clinical cases reported was 14.0% (n = 458). Unadjusted analysis showed significant differences in pregnancy (6% in nonpregnant cows vs. 15% in pregnant cows) and parity (9% in first-parity cows, 16% in second- and third-parity cows, and 18% in cows with ≥4 parities). Across all 12 pens, the proportion of clinical cases ranged from 7% to 27%, and when restricted to comparable pens (n = 5), the variation was similar (11% to 24%). The matched odds ratio (OR), using nonpregnant cows as the reference group, was 4.9 (95% CI: 1.6–14.9), indicating higher odds of being a case among pregnant cows. Among pregnant cows, the OR for multiparous cows versus primiparous cows was 2.1 (95% CI: 1.5–2.8). The findings suggest that pregnancy and parity are 2 cow-level factors that contribute to HPAI H5N1 clinical disease. Expanding the number of herds studied is critical for a better understanding of these risk factors.

## Highlights

• Pregnant cows had 4.9 times higher odds of clinical disease than nonpregnant cows.
• Multiparous cows had 2.1 times higher odds of clinical disease than first-parity cows.
• Clinical disease varied widely across pens housing cows with similar characteristics.

## Graphical Abstract

**Figure:**
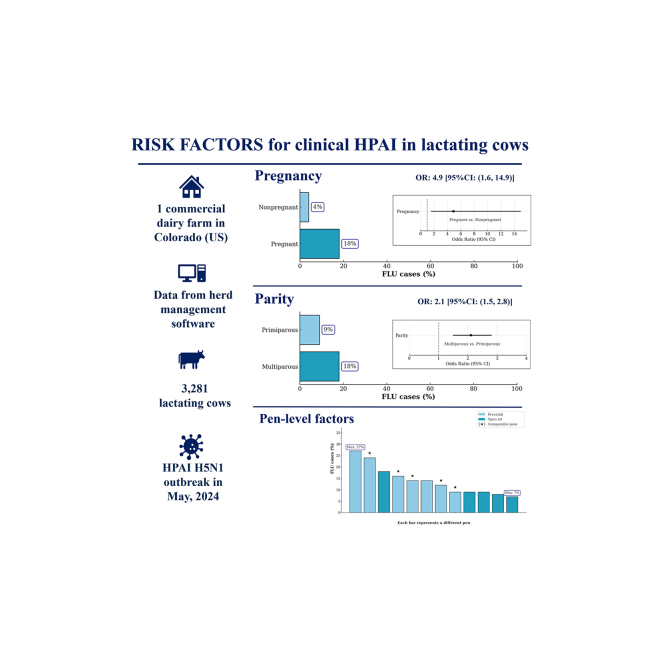
**Summary:** This observational study examined cow-level risk factors associated with clinical highly pathogenic avian influenza (HPAI) H5N1 in lactating Holsteins during a May 2024 outbreak on a dairy farm in Colorado. Using herd records, the study aimed to characterize clinical cases, evaluate the association between pregnancy and clinical disease, and identify risk factors specific to pregnant cows. Overall, 14.0% of the herd developed clinical disease, with incidence varying across pens (ranging from 7% to 27%), even among those housing cows with similar characteristics. Pregnant cows were 4.9 times more likely to develop clinical disease compared with nonpregnant cows, whereas cows with multiple lactations had 2.1 times greater odds compared with first-lactation cows. These results suggest that pregnancy status, parity, and pen-level factors may play an important role in determining susceptibility to clinical disease during an HPAI outbreak in dairy cattle.

The highly pathogenic avian influenza (**HPAI**) virus H5N1 clade 2.3.4.4b was first detected in wild migratory waterfowl in the United States in 2021, following widespread outbreaks across Asia and Europe (Bevins et al., 2022). Since its introduction, this virus has caused important outbreaks in wild bird populations and severe economic disruptions within the poultry industry (Sambucci et al., 2025; USDA-APHIS, 2025).

In March 2024, the first spillover of HPAI H5N1 to dairy cattle was confirmed on a Texas dairy farm after unexplained dramatic declines in milk production (Nguyen et al., 2025). Molecular assays conducted by the USDA confirmed wild bird to cattle transmission, marking an unprecedented cross-species transmission event (Nguyen et al., 2025). As of April 5, 2025, outbreaks of HPAI H5N1 in dairy cattle have affected 998 herds across 17 states (USDA-APHIS, 2025).

The mechanisms facilitating the introduction of HPAI H5N1 into dairy herds remain unclear. Epidemiological evidence suggests that interstate movement of infected dairy cattle has contributed significantly to the spread (Caserta et al., 2024; Nguyen et al., 2025). Additionally, it is suspected that farm-to-farm transmission occurs through routine farm activities, including the movement of personnel, vehicles, and equipment between affected and unaffected premises (USDA-APHIS, 2025). However, the rapid progression of outbreaks suggests other transmission pathways may exist, requiring further investigation.

Once introduced into a herd, the primary cow-to-cow transmission routes are not fully characterized. Although influenza A viruses typically target respiratory tissues, the extent of direct or indirect respiratory transmission in dairy cattle remains unclear (Butt et al., 2024). The novel HPAI H5N1 virus demonstrated a strong affinity for mammary tissue; experimental infection via intramammary inoculation has produced clinical infection (Baker et al., 2024; Caserta et al., 2024; Halwe et al., 2025). It has been suggested that contaminated milking equipment, bedding, or direct contact with infected milk could facilitate cow-to-cow transmission (Butt et al., 2024). Moreover, the role of contaminated environments remains uncertain.

Clinical manifestations of HPAI H5N1 infection in dairy cattle include abnormal milk (thick, colostrum-like), drastic reductions in milk production, dehydration, nasal discharge, lethargy, and gastrointestinal disturbances characterized by tacky or loose feces (Burrough et al., 2024; Caserta et al., 2024; Oguzie et al., 2024). Disease severity varies considerably among affected herds, with reported morbidity rates ranging from 3% to 20% (Caserta et al., 2024), and 10% to 15% (Burrough et al., 2024; Rodriguez et al., 2024). However, personal communication with dairy producers in California suggests that actual morbidity may be higher than in previous published estimates. Moreover, a study conducted by Peña-Mosca et al. (2025) on a farm in Ohio found a herd-level seroprevalence of 89.4%, with 76.1% of seropositive cows showing no clinical signs, indicating subclinical infection. Determining whether specific cow-level factors predispose animals to clinical illness could provide critical insights for targeted herd management and improved biosecurity measures (Lombard and Garry, 2025).

This observational study aims to examine cow-level factors such as DIM, milk yield, parity, pregnancy, and days carrying calf (**DCC**) to identify risk factors influencing the manifestation of clinical HPAI H5N1 infections using on-farm herd records from a single dairy farm. Specifically, the objectives were to (1) describe characteristics of cows identified as HPAI H5N1 clinical cases, (2) evaluate the relationship between pregnancy status and the manifestation of clinical disease, and (3) identify additional risk factors associated with the clinical disease in pregnant cows.

Herd records were obtained from DairyComp305 herd management software (Valley Agricultural Software, Tulare, CA) from a commercial dairy farm in Colorado. The farm experienced an outbreak of HPAI H5N1, with peak clinical signs during the last week of May 2024. The dairy operated 2 nearby facilities (<3.5 km apart), collectively housing more than 7,000 lactating cows. Cow placement at these facilities depended on lactation stage and pregnancy status; early lactation cows were managed at location 1, and mid-to-late lactation cows were housed at location 2.

Only cows from location 2 were included in the current analysis, as only this location had daily milk yield records. The study population consisted of 3,281 lactating Holstein cows present on May 1, 2024, before detection of the outbreak, excluding cows located in hospital pens. These cows were housed in 12 pens, including 7 freestall pens (indoor housing with individual stalls; n = 2,126) and 5 open-lot pens (outdoor housing with shared open space; n = 1,155).

The herd veterinarian (B.N.) led the outbreak response, focusing on 3 main strategies: (1) minimizing disruptions to routine herd management practices; (2) selectively treating cows exhibiting one or more clinical signs such as reduced milk yield, colostrum-like milk, severe dehydration, or anorexia; and (3) systematically recording treated cows. Clinical cases were identified based on entries in the herd management system under a newly introduced code, **FLU**, which was assigned to cows that required supportive treatment. This code did not specify the exact date of clinical onset. The FLU code referred to a binary variable, coded as 0 if no treatment for clinical disease was administered, and 1 if treatment was given. In this study, clinical cases were defined as cows with a FLU event recorded by August 31, 2024, the date on which this information was extracted.

Other data extracted included cow-level variables such as parity, DIM, milk yield, pregnancy, DCC, and pen ID. All cow-level information was obtained from records dated May 1, 2024, except for daily milk yield. Milk yield was calculated as the 7-d average from April 25 to May 1, 2024.

Disease frequency was measured as the proportion of FLU cases, interpreted as a proxy for incidence. The numerator included cows identified as clinical cases (cows present in the herd on May 1 that recorded as FLU cases by August 31, 2024). The denominator comprised all cows, regardless of case status (cows present in the herd on May 1, 2024).

All statistical analyses were performed using the R statistical programming environment (version 4.3.2; https://www.r-project.org), with significance declared at *P* ≤ 0.05.

Initially, univariate analyses were conducted to identify potential variables of interest. To evaluate differences in FLU status, a *t*-test was used to compare mean differences in continuous variables (DIM, milk yield, and DCC), whereas the chi-squared test of independence was used for categorical variables (parity, and pregnancy). Furthermore, the proportion of FLU cases within each pen was reported descriptively to provide preliminary insights into pen-level variability.

To examine if differences in the clinical manifestation of the disease were observed between pregnant and nonpregnant cows, a matched case-control analysis was conducted to control for potential confounders, based on the assumption that some cows in the study population differed only in their pregnancy status. Pregnant cows were individually matched 1:1 to nonpregnant cows based on parity, lactation stage (100–200 DIM, 200–300 DIM, and >300 DIM), milk yield categories (<20 L/d, 20–30 L/d, 30–40 L/d, and > 40 L/d), and pen ID. Only pregnant cows that conceived before 150 DIM (cows that did not experience delayed conception) and nonpregnant cows within 128–375 DIM were included to ensure comparability between groups by keeping both within a similar DIM range. Conditional logistic regression was applied using the Epi package in R (Carstensen et al., 2024), with FLU status as the dependent variable and pregnancy as the independent variable. The matched odds ratio (**OR**) served as the measure of association. Additionally, the proportion of FLU cases was reported separately for pregnant and nonpregnant cows.

To identify specific risk factors associated with FLU among pregnant cows, the analysis was restricted to those cows that conceived before 150 DIM (cows that did not experience delayed conception) and were housed in comparable pens. Pens were classified as comparable if they shared the same facility design (freestall or open lot) and if they housed cows with similar distributions of cow-level characteristics (parity, DIM, milk yield, pregnancy status, and DCC). To identify pens with comparable cow-level characteristics ANOVA (DIM, milk yield, DCC) and chi-squared tests of independence (parity and pregnancy status) were used. Post hoc pairwise comparisons were performed using Tukey's honestly significant difference tests for continuous variables and chi-squared tests with Bonferroni adjustments for categorical variables. Pens were considered similar for a given variable if no significant differences (*P* < 0.05) were found in pairwise comparisons. A network-based clustering approach was applied to each variable using the rcompanion and igraph packages in R (Csárdi et al., 2025; Mangiafico, 2025). Pens were represented as nodes, with edges drawn between pens that showed no significant pairwise differences. Pens were considered comparable if they clustered together across all network analyses. Using this subset, the outcome FLU was modeled using a mixed-effects logistic regression with parity, DIM, DCC, and milk yield as fixed effects, and pen ID as a random effect, implemented via the lme4 package in R (Bates et al., 2015). If continuous predictors did not meet the assumption of linearity with the outcome on the logit scale, as assessed visually after dividing the predictor into equal-sized bins (Hosmer et al., 2013), they were categorized according to industry standards. Categories were established for DIM as mid lactation (100–200 DIM), late lactation (200–300 DIM), and extended lactation (>300 DIM); for DCC as early (<90 DCC), mid (90–180 DCC), and late gestation (>180 DCC); and for milk yield relative to the herd average as low (<75%), average, or high (>125%). Cows with 2 or more parities were grouped together into a single category. Variables were initially screened using a liberal significance threshold (*P* ≤ 0.20), followed by backward selection based on statistical significance and a 20% change-in-estimate criterion. Model fit was assessed using Akaike information criterion (**AIC**) and the likelihood ratio test. The OR and proportions of FLU cases were reported for variables retained in the final model.

As of May 1, 2024, the herd at location 2, excluding cows located in hospital pens, consisted of 3,281 cows. Most cows were over 200 DIM (75.0%) and pregnant (90.6%), with DCC ranging from 60 to 225 d. By August 31, 2024, 458 cows (14.0%) had been identified as clinical cases. The characteristics of the full study population based on FLU status are described in Table 1. Based on the univariate analysis, no significant differences were observed across FLU status for DIM (*P* = 0.15), DCC (*P* = 0.39), and milk yield (*P* = 0.55). However, pregnancy (*P* < 0.001) and parity (*P* < 0.001) showed significant differences. The crude proportion of FLU cases was 6% in nonpregnant cows and 15% in pregnant cows. Similarly, it was 9% in first-parity cows, increasing to 16% in second- and third-parity cows and 18% in cows with 4 or more parities. The distribution of FLU cases varied across the 12 pens in location 2 (range: 7% to 27%); this variation was also observed across the 5 pens that were identified as comparable based on cow-level factors and facility type (11%–24%; Figure 1).

**Table 1:** Description of the cows included in this study conducted on a commercial dairy farm in Colorado, which experienced an HPAI H5N1 outbreak in the last week of May 2024^1^

Full study population	Total	Non-cases^2^	Cases^2^	*P*-value^3^
Cows (n)	3,281 (100%)	2,823 (86.0%)	458 (14.0%)	NA
Parity^4^				
1	1,222 (37.2%)	1,106 (39.2%)	116 (25.3%)	<0.001
2	821 (25.0%)	690 (24.4%)	131 (28.6%)	
3	611 (18.6%)	511 (18.1%)	100 (21.8%)	
4+	627 (19.2%)	516 (18.2%)	111 (24.2%)	
DIM^4^ (d)	250	250	255	0.15
Pregnant cows^4^	2,974 (90.6%)	2,533 (89.7%)	441 (96.3%)	<0.001
Days carrying calf^4^ (d)	160	160	163	0.39
Milk yield^4^, ^5^ (L/d)	33.7	33.7	33.9	0.55

Pregnancy-based matched subset	Total	Nonpregnant^4^	Pregnant^4^	*P*-value^3^
Cows (n)	196 (100%)	98 (50.0%)	98 (50.0%)	1.00
Parity^4^ (n)				
1	60 (30.6%)	30 (30.6%)	30 (30.6%)	1.00
2	48 (24.5%)	24 (24.5%)	24 (24.5%)	
3	50 (25.5%)	25 (25.5%)	25 (25.5%)	
4+	38 (19.4%)	19 (19.4%)	19 (19.4%)	
DIM^4^ (d)	266	268	264	0.62
Days carrying calf^4^ (d)	NA	NA	167	NA
Milk yield^4^, ^5^ (L/d)	28.7	29.0	28.4	0.20
Cases^1^	22 (11.2%)	4 (4.1%)	18 (18.4%)	0.001

Pregnant cow subset	Total	Non-cases^2^	Cases^2^	*P*-value^3^
Cows (n)	1,556	1,329 (85.4%)	227 (14.6%)	NA
Parity^4^ (n)				
1	60 (30.6%)	589 (44.3%)	61 (26.9%)	<0.001
2	48 (24.5%)	335 (25.2%)	67 (29.5%)	
3	50 (25.5%)	215 (16.2%)	52 (22.9%)	
4+	38 (19.4%)	190 (14.3%)	47 (20.7%)	
DIM^4^ (d)	251	251	251	0.98
Days carrying calf^4^ (d)	144	144	144	0.98
Milk yield^4^, ^5^ (L/d)	34.6	34.5	34.7	0.32

1 The full study population consisted of all lactating cows present on the farm as of May 1, 2024, excluding those located in the hospital pens. Two analytical subsets were created: (1) a pregnancy-based matched subset, created under the assumption that pregnancy was the only differing factor between pregnant and nonpregnant cows, and (2) a pregnant cow subset, which included only cows that conceived before 150 DIM and were housed in “comparable” pens. Pens were considered comparable based on similar cow-level characteristics (parity, DIM, milk yield, pregnancy status, and days carrying calf) and facility type.

2 Clinical case and non-cases of HPAI H5N1 based on herd records on August 31, 2024.

3 *P*-values were obtained using Student's *t*-test, chi-squared test of independence, and 2-proportion z-test. NA = not applicable.

4 Cow-level data were obtained from records dated May 1, 2024.

5 Seven-day average daily milk yield (L/d), calculated from April 25 to May 1, 2024.

**Figure 1:**
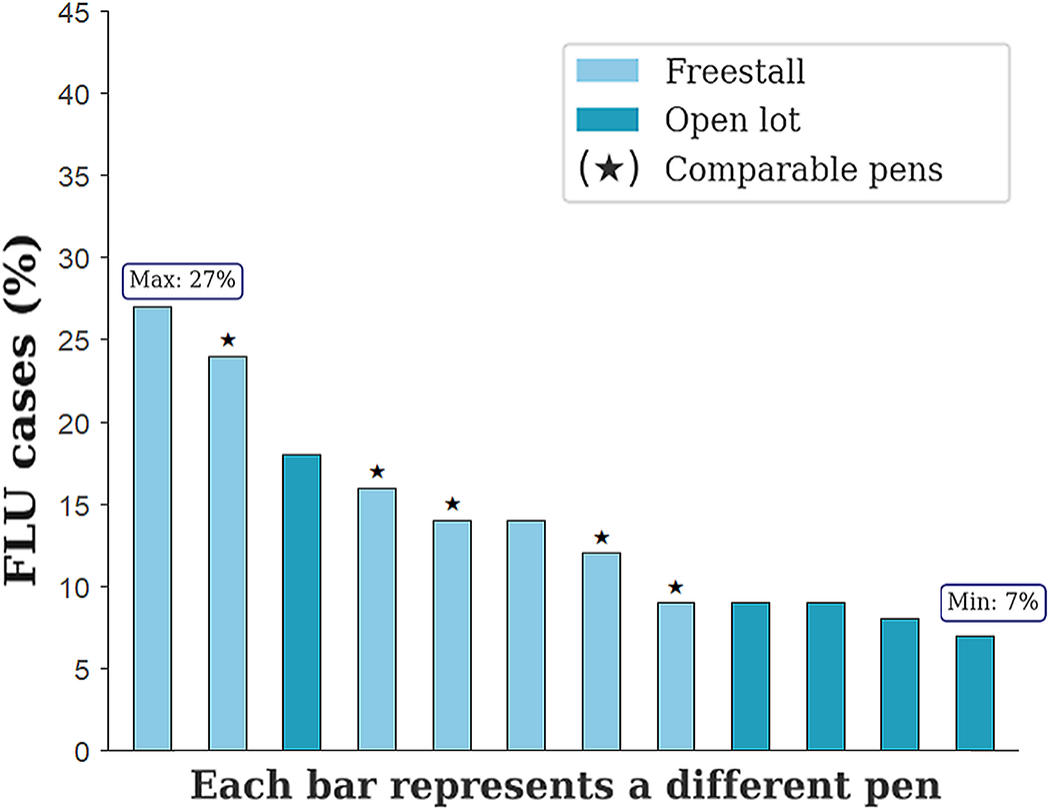
Bar plot showing the proportion of FLU cases (n = 458), defined as cows treated for clinical HPAI H5N1, across the pens (n = 12) housing lactating cows (n = 3,281) in a commercial dairy farm in Colorado. Bar colors indicate facility design: freestall (indoor housing with individual stalls) or open lot (outdoor housing with shared open space). Pens identified as comparable based on cow-level characteristics (parity, DIM, milk yield, pregnancy status, and DCC) and facility type are marked with a star. Max = maximum; Min = minimum.

The pregnancy-based matched subset used to evaluate the relationship between pregnancy and FLU events (aim 2) included 196 cows (98 pregnant, 98 nonpregnant) with an average parity of 2.5, 266 DIM, and 167 DCC (Table 1). The observed proportion of FLU cases in this group was 4.1% in nonpregnant cows and 18.4% in pregnant cows (Figure 2A). The matched OR, using nonpregnant cows as the reference group, was 4.9 (95% CI: 1.6–14.9).

**Figure 2:**
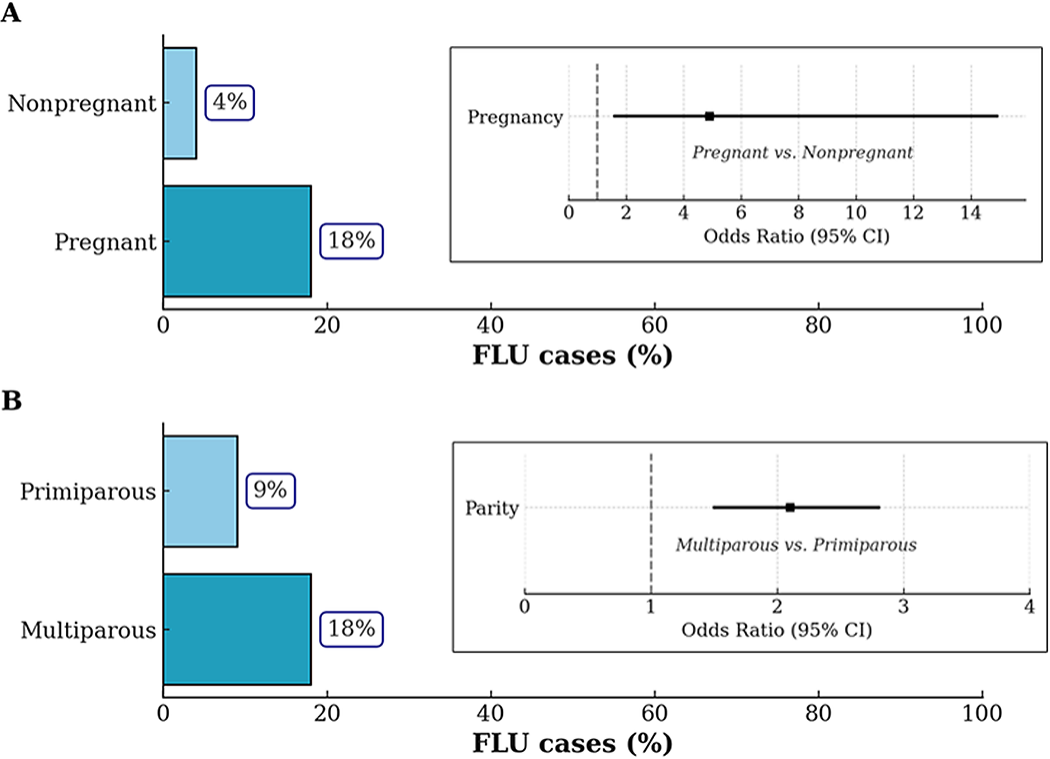
Bar plots showing the proportion of FLU cases, defined as cows treated for clinical HPAI H5N1, along with confidence interval lines representing the measure of association for 2 specific risk factors in a commercial dairy farm in Colorado. Pregnancy (A) was analyzed using a matched subset (n = 196), where pregnant cows were individually matched 1:1 to nonpregnant cows based on parity, lactation stage, milk yield categories, and pen ID. Parity (B) was analyzed using a subset of pregnant cows (n = 1,556) that conceived before 150 DIM and were housed in comparable pens. Pens were considered comparable if they shared the same facility type (freestall or open lot) and exhibited similar distributions of cow-level characteristics, including parity, DIM, milk yield, pregnancy status, and days carrying calf.

The subset of pregnant cows (n = 1,556) used to identify cow-level risk factors is described in Table 1. The best-fitting model included parity, categorized as primiparous vs. multiparous, as the only predictor. The final model showed improved fit compared with the null model, as indicated by a lower AIC (1,261.6 vs. 1,280.4) and a significant likelihood ratio test (*P* < 0.001). The observed proportion of FLU cases in this subset was 9.0% in primiparous cows and 18.0% in cows with 2 or more parities (Figure 2B). The OR, using first-parity cows as the reference group, was 2.1 (95% CI: 1.5–2.8).

One year after the first confirmed case of HPAI H5N1 in dairy cattle (Texas, March 24, 2024), many aspects of this emerging disease remain poorly understood. Identifying cow-level risk factors for disease severity is a crucial step toward improving biosecurity practices and preventing exposure of susceptible animals to the virus (Lombard and Garry, 2025). Such knowledge can also provide valuable insights into the mechanisms influencing disease progression, especially among high-risk groups. In this study, we investigated cow-level factors associated with the occurrence of clinical signs during an outbreak on a commercial dairy farm in Colorado using data from herd records. Among the variables analyzed, only pregnancy and parity were significantly associated with clinical disease.

The proportion of animals reported as FLU cases in our study was within the range of previous studies that ranged from 3% to 20% (Caserta et al., 2024), and 10% to 15% (Burrough et al., 2024; Rodriguez et al., 2024). At present, there are no standardized protocols to define clinical cases of HPAI. In our study, farm management classified cows as clinical cases if they required supportive treatment based on empty udders at milking, colostrum-like milk, severe dehydration, and anorexia. Records were systematically maintained for all treated animals, following a proactive decision by the dairy management team before the outbreak to ensure comprehensive documentation, particularly to support potential HPAI H5N1-related USDA funding opportunities.

Our finding that pregnancy is associated with higher odds of being classified as a clinical case has not been previously reported in the literature. However, Burrough et al. (2024) and Peña-Mosca et al. (2025) observed that cows in mid-to-late lactation were more frequently affected by clinical HPAI H5N1 compared with those in early lactation. However, their analyses did not account for the potential confounding effect of pregnancy status on lactation stage. The association between pregnancy and clinical disease presentation is biologically plausible, as pregnancy induces immunological adaptations that promote fetal tolerance but can compromise maternal immune defenses (Creisher and Klein, 2024). Additionally, elevated progesterone and estrogen levels during pregnancy are known to modulate immune function, potentially increasing susceptibility to infections (Cervantes et al., 2022).

Consistent with our findings, previous studies have reported that parity is a cow-level risk factor associated with HPAI H5N1 infection (Burrough et al., 2024; Peña-Mosca et al., 2025). Although the mechanisms underlying increased susceptibility in multiparous cows remain unclear, immunological, metabolic, and physiological factors are likely contributors (Lean et al., 2023). Recent research indicates that older cows often display signs of chronic immune activation, including elevated expression of immune defense genes, which is indicative of immunosenescence (Buggiotti et al., 2021). This sustained immune activation may impair the ability to mount effective responses to novel pathogens such as H5N1 and is further compounded by a proinflammatory baseline state that can weaken immune defenses (Buggiotti et al., 2021). Additionally, parity-related differences in immune energy use have been noted: primiparous cows favor β-oxidation, whereas multiparous cows shift toward glycolysis, reflecting higher metabolic stress. Along with age-related IGF-I decline, this may impair immunity and raise infection risk in multiparous cows (Ruprechter et al., 2020).

Interestingly, one of the most common diseases in dairy cows, mastitis, shows a similar pattern to HPAI H5N1 in terms of stage of lactation and parity, with higher occurrence in multiparous cows during mid-to-late lactation (Chen et al., 2023). Given the strong mammary tropism of H5N1 (Baker et al., 2024; Caserta et al., 2024; Halwe et al., 2025), the interplay between HPAI H5N1 disease, udder health, and pregnancy should be further explored. However, Peña-Mosca et al. (2025) initially found no differences in somatic cell counts between cows with clinical HPAI H5N1 and those without.

In our study, the proportion of FLU cases varied considerably across pens, even among those considered comparable (freestall facilities housing cows with similar characteristics). This suggests that pen-level factors warrant further investigation as potential risk factors influencing susceptibility to clinical disease. If similar within-farm differences are observed in other herds, factors such as pen orientation, overcrowding, milking order, feeding sequence, and others should be explored as factors that affect the manifestation of clinical signs.

A limitation of this study is that our findings are based on a single outbreak, which restricts the external validity and generalizability of the results. Odds ratio was used as the measure of association, and the results were interpreted accordingly. Although the outcome was relatively common (14% clinical cases) and OR may overestimate the strength of the association compared with risk ratios, the direction of the effect is generally consistent (Dohoo et al., 2012). Additionally, the duration of clinical cases and treatments administered were not recorded at the individual cow level, as the dairy's operational team was overwhelmed during the outbreak. Although the clinical signs used on-farm to identify suspected influenza cases aligned with those described by Caserta et al. (2024), the absence of standardized diagnostic procedures introduces potential subjectivity. Furthermore, potential misclassification bias may have occurred due to the disease definition being based on producer-reported clinical signs, and selection bias is also possible because only cows that received treatment were included. Future research should aim to reduce this limitation by implementing consistent diagnostic protocols and structured recording systems, as automated sensor technologies have shown promise in detecting health issues in dairy cattle, including during HPAI outbreaks (Rodriguez et al., 2025).

In summary, pregnancy and parity were identified as cow-level factors significantly associated with clinical disease, suggesting that physiological and immunological changes linked to these states may increase susceptibility to HPAI H5N1. Additionally, our findings indicate that pen-level factors warrant further investigation as potential contributors to disease risk. Future research should focus on identifying other cow-level characteristics that may predispose animals to clinical disease. This knowledge could support the development of targeted management strategies during outbreaks, including enhanced monitoring of high-risk groups, stricter biosecurity measures, and timely supportive interventions to mitigate the impact of HPAI H5N1 on dairy herds.
